# Uridine from *Pleurotus giganteus* and Its Neurite Outgrowth Stimulatory Effects with Underlying Mechanism

**DOI:** 10.1371/journal.pone.0143004

**Published:** 2015-11-13

**Authors:** Chia-Wei Phan, Pamela David, Kah-Hui Wong, Murali Naidu, Vikineswary Sabaratnam

**Affiliations:** 1 Mushroom Research Centre, Institute of Biological Sciences, Faculty of Science, University of Malaya, 50603 Kuala Lumpur, Malaysia; 2 Centre of Excellence for Learning and Teaching, UCSI University, No. 1, Jalan Menara Gading, UCSI Heights, 56000 Cheras, Kuala Lumpur, Malaysia; 3 Department of Anatomy, Faculty of Medicine, University of Malaya, Kuala Lumpur, Malaysia; 4 Institute of Biological Sciences, Faculty of Science, University of Malaya, Kuala Lumpur, Malaysia; University of Pecs Medical School, HUNGARY

## Abstract

Neurodegenerative diseases are linked to neuronal cell death and impairment of neurite outgrowth. An edible mushroom, *Pleurotus* giganteus was found to stimulate neurite outgrowth *in vitro* but the chemical constituents and the underlying mechanism is yet to be elucidated. The chemical constituents of *P*. *giganteus* (linoleic acid, oleic acid, cinnamic acid, caffeic acid, *p*-coumaric acid, succinic acid, benzoic acid, and uridine) were tested for neurite outgrowth activity. Uridine (100 μM) was found to increase the percentage of neurite-bearing cells of differentiating neuroblastoma (N2a) cells by 43.1±0.5%, which was 1.8-fold higher than NGF (50 ng/mL)-treated cells. Uridine which was present in *P*. *giganteus* (1.80±0.03 g/100g mushroom extract) increased the phosphorylation of extracellular-signal regulated kinases (ERKs) and protein kinase B (Akt). Further, phosphorylation of the mammalian target of rapamycin (mTOR) was also increased. MEK/ERK and PI3K-Akt-mTOR further induced phosphorylation of cAMP-response element binding protein (CREB) and expression of growth associated protein 43 (GAP43); all of which promoted neurite outgrowth of N2a cells. This study demonstrated that *P*. *giganteus* may enhance neurite outgrowth and one of the key bioactive molecules responsible for neurite outgrowth is uridine.

## Introduction

The economic burden of neurodegenerative disease is enormous and is expected to grow rapidly as more people live longer. Current drug therapy for neurodegenerative diseases is ineffective with many side effects and it only provides a short term delay in the progression of the disease. Further, the drug development pipeline is drying up and the number of drugs reaching the market has lagged behind the growing need for such drugs. It is therefore of utmost importance to find appropriate solutions to prevent, or perhaps impede, the development of neurodegenerative diseases.

An alternative approach to prevent or treat such diseases is by dietary supplementations and functional foods. Functional food is food that has a potentially positive effect on health beyond basic nutrition and it is considered to offer additional benefits that may reduce the risk of disease or promote optimal health [1]. Trials with nerve growth factor (NGF) for Alzheimer's disease had gained some degree of success but the high molecular weight of NGF reduces its ability to cross the blood-brain barrier [2]. Considering the limitation of the above, early intervention using mushrooms as functional food may be a helpful strategy. Mushrooms have long been celebrated as a source of powerful nutrients. The polysaccharides found in mushrooms have been described as effective immuno-modulating agents [3]. Mushrooms are especially rich in vitamin D_2_ when exposed to UV light and it was found that vitamin D_2_-enriched white button mushroom improved the memory of Alzheimer’s transgenic mice [4,5]. Several compounds isolated from mushrooms have been shown to promote neurite outgrowth, for example, hericenones and erinacines from the lion’s mane mushroom, *Hericium erinaceus* (Bull.: Fr.) Pers [6,7]. Other mushrooms found to trigger neurite outgrowth are *Ganoderma lucidum* (Fr) P. Karst, *Lignosus rhinocerotis* (Cooke) Ryvarden, *Ganoderma neo-japonicum* (Fr) P. Karst, and *Cordyceps militaris* (L.:Fr.) Link [8]. These mushrooms are able to exert neuroprotective effects and promote neuritogenesis as well as play a role in neuroregeneration.

*Pleurotus giganteus* (Berk.) Karunarathna & K.D. Hyde is one of the edible mushrooms which have been shown to exert neurite outgrowth stimulatory effects [9,10]. The nutritional composition of the mushroom exhibits high antioxidant activities [11]. However, there is a need to elucidate the chemical compounds which contribute to the neuritogenic properties of this mushroom. Therefore, in this study, the efficacy of the individual chemical constituents of *P*. *giganteus* in stimulating neurite outgrowth of N2a cells was investigated. The chemical constituents selected were linoleic acid, oleic acid, cinnamic acid, caffeic acid, *p*-coumaric acid, succinic acid, benzoic acid, and uridine based on a previous study [12]. In order to confirm the underlying mechanisms of the neurite outgrowth activity induced by the chemical compound and the standardised mushroom extracts, treatment of cells with specific inhibitors was carried out followed by measurement of the phosphorylated kinases using enzyme-linked immunosorbent assays.

## Materials and Methods

### Materials

**Fig 1:**
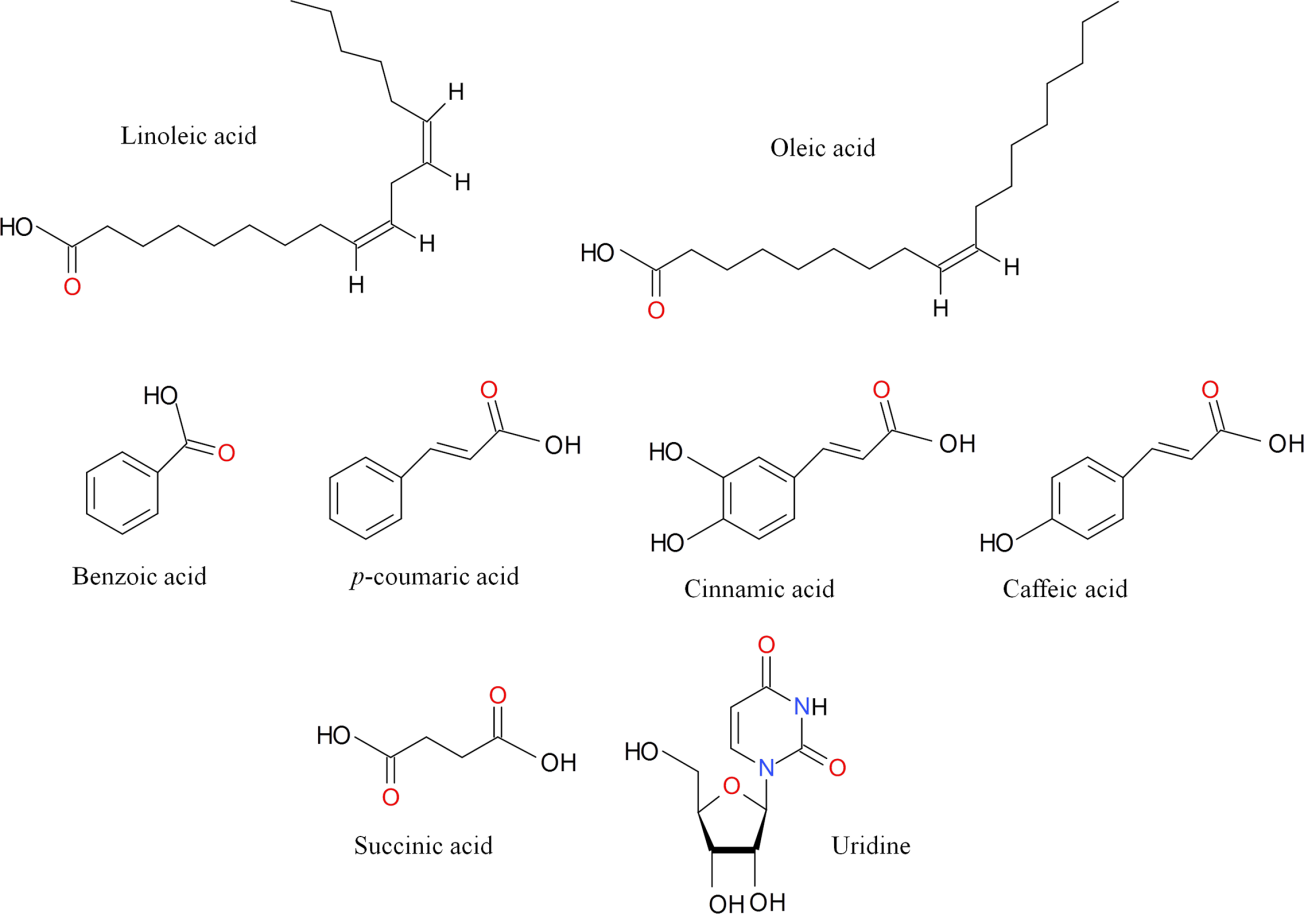
Compounds detected in the basidiocarps of *P*. *giganteus*.

Nerve growth factor (NGF), U0126, PD98059, LY294002, suramin, pyridoxal phosphate-6-azophenyl-2'4'-disulfonic acid (PPADS) were purchased from Sigma (St. Louis, MO, USA). Ethanol and methanol were purchased from Merck (Germany). Linoleic acid, oleic acid, cinnamic acid, caffeic acid, *p*-coumaric acid, succinic acid, benzoic acid, and uridine were previously detected in the basidiocarps of *P*. *giganteus* using GCMS and LCMS/MS (Fig 1) [12]. All the compounds were purchased from Sigma (St. Louis, MO, USA).

### Cell culture

Mouse neuroblastoma cells (N2a) was purchased from American Type Culture Collection (ATCC, USA). N2a cells were cultured in Eagle’s minimum essential medium (MEM) with L-glutamine (PAA) supplemented with 10% (v/v) heat-inactivated foetal bovine serum (PAA), 100 U/ml penicillin, and 100 μg/ml streptomycin. All the cells were maintained at 37°C and 5% CO_2_ in a humidified atmosphere. The cells were subcultured at 3–4 days intervals.

### Preparation of mushroom extracts

The fresh basidiocarps of *P*. *giganteus* were freeze-dried and ground to powder. To obtain an aqueous extract, the freeze dried mushroom powder was soaked in distilled water (1:20, w/v) at room temperature in a shaker (200 rpm) for 24 h. The mixture was double boiled in water bath for 30 min, cooled, and then filtered (Whatman No. 4). The resulting aqueous extract was freeze-dried and kept at -20°C prior to use. For ethanol extraction, the freeze dried powder was soaked in 95% ethanol at room temperature. The extracted ethanol solution was vacuum-evaporated (rotary evaporator Eyela N-1000, USA) for further uses.

### Neurite outgrowth assay (quantification of neurite bearing cells and chromogenic method)

N2a cells were seeded in a 24-well culture plate at an initial density of 5,000 cells per well containing complete growth medium (1 mL/well) and incubated overnight. To induce cell differentiation, the complete medium was carefully replaced with 5% serum medium before exposure to mushroom extracts or compounds. NGF-treated cell was used as a positive control while cells with medium only served as a negative control. All the cells were incubated for 48 h at 37°C, 95% air and 5% CO_2_ to observe neuritogenic activity, if any. Five random fields (100–200 cells) were examined in each well by using a phase contrast microscope at 20× magnifications equipped with QImaging Go-3 camera (QImaging, Canada). Neurite length was measured in at least 30 cells in randomly chosen fields by using image processor system Image-Pro Insight (MediaCybernetics, MD). The number of neurite outgrowths, defined as axon-like extensions that are double or more than the length of the cell body diameter was recorded [13]. The percentage of neurite bearing cells (%) is the number of neurite bearing cells divided by the total number of cells in a field and then multiplied by 100%. For neurite outgrowth assay using chromogenic method, the NS220 Neurite Outgrowth Assay Kit (Merck Millipore, Germany) was used following the protocol of the manufacturer.

### Quantification of uridine in *P*. *giganteus* extracts

Uridine reference compound was accurately weighed and dissolved in water to prepare the standard chemical solutions (1 mg/mL). Perkin Elmer Series 200 liquid chromatography equipped with a Perkin Elmer Series 200 UV detector was used. The detector signal was recorded by the Turbochrom workstation software. The column used was Hypersil BDS C18 (4.6 × 250 mm) with Alltech refillable C18 Guard column (10 × 4.6 mm) (Alltech, USA). The mobile phase consisted of methanol: 10 mM monobasic potassium phosphate (15:85; pH 5.0) and the flow rate was 1.5 mL/min. The calibration curve was prepared by injecting a series of uridine standard dilutions. Uridine in extracts was quantified by means of calibration curves obtained from standard from Sigma.

### Treatment with specific inhibitors

Stock solution (10 mM) of mitogen extracellular signal-regulated kinase (MEK) inhibitors (U0126, PD98059) and phosphoinositide 3-kinase (PI3K) inhibitor (LY294002) were prepared in DMSO and stored in −20°C in the dark. Each inhibitor i.e. U0126 (10 μM), PD98059 (40 μM), and LY294002 (30 μM) was then prepared by diluting in medium prior to use. Purinergic 2Y (P2Y) receptor antagonists, suramin (30 μM) and pyridoxalphosphate-6-azophenyl-2',4'-disulfonic acid (PPADS; 30 μM) were prepared in double distilled water and protected from light. Cells were either incubated with or without the treatment of inhibitors for one hour prior to bioassays.

### Measurement of signaling pathway and neuronal marker proteins

Mushroom extracts or compound was added to N2a cells with or without pretreatment with specific inhibitors. The cells were harvested and cell lysates were prepared. The cell lysates were then incubated in microwells pre-coated individually with phosphorylated extracellular signal-regulated kinase (p-ERK; Thr202/Tyr204), phosphorylated protein kinase B (p-Akt; Thr308), p-MEK1 (Ser217/221), phosphorylated mammalian target of rapamycin (p-mTOR; Ser2448), or phosphorylated cAMP-response element binding protein (p-CREB; Ser133) antibodies. To increase sensitivity, the plate was incubated overnight at 4°C. The wells were then washed four times with washing buffer (10 mM phosphate buffer pH 7.4, 150 mM NaCl, and 0.05% Tween 20). Following washing, detection antibody was added (Cell Signaling Technology, Inc). Anti-rabbit IgG, horseradish peroxidase (HRP)-linked antibody was then added to recognise the bound detection antibody. HRP substrate, which is TMB, was then added for colour development. Absorbance at 450 nm was recorded and the magnitude of absorbance is proportional to the quantity of the p-ERK, p-Akt, p-MEK1, p-mTOR, and p-CREB antibodies. Similarly, the levels of GAP-43, TUBA4A, and TUBb1were determined from a standard curve plotted with known concentrations of the respective proteins.

### Statistical Analysis

All the experimental data are expressed as mean ± standard deviation (S.D). Statistical differences between groups were analysed and calculated by one-way analysis of variance (ANOVA) from at least three independent experiments. This was followed by Duncan's multiple range tests. *P* < 0.05 was considered to be significant between groups. The EC_50_ of uridine which is the concentration that yielded half-maximal neurite outgrowth stimulatory activity was calculated by Prism 6 software (Graphpad Software, Inc., USA).

## Results

### The effects of different chemical constituents of *P*. *giganteus* on neurite outgrowth activity

**Fig 2:**
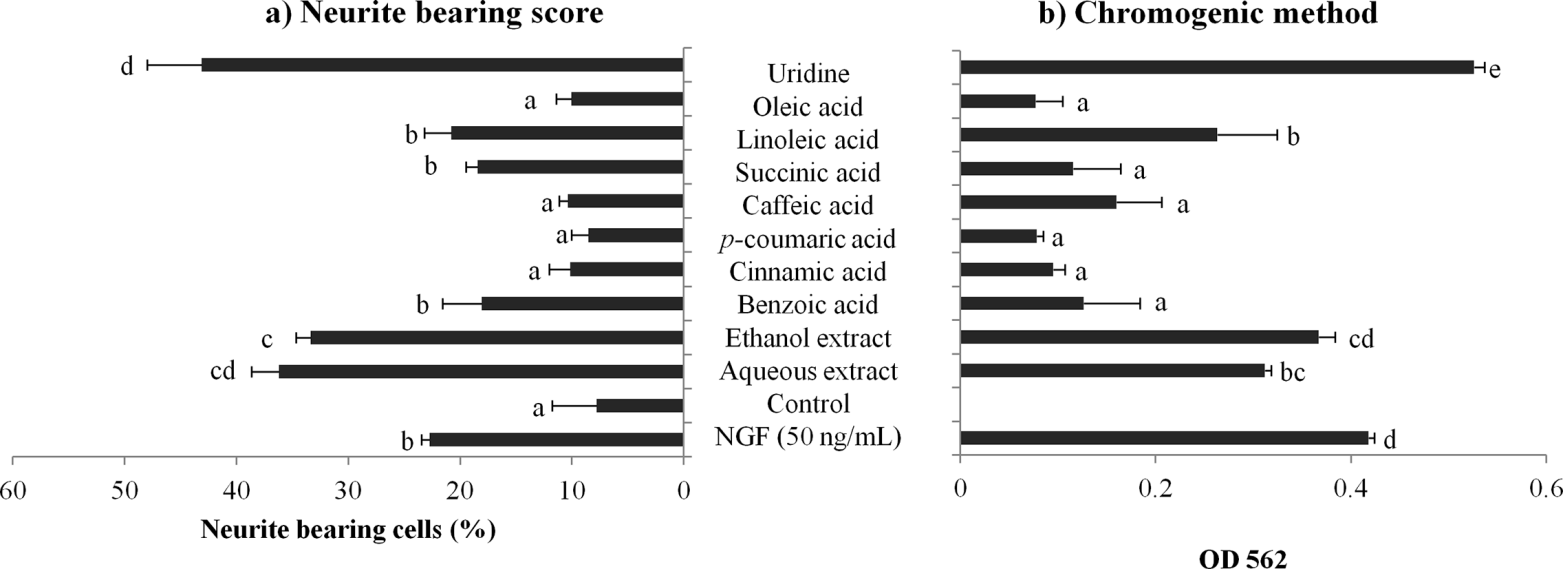
The effects of mushroom extracts and compounds on neurite outgrowth of differentiating N2a cells by using (a) quantification of neurite bearing cells, and (b) chromogenic method at optical density 562. NGF (50 ng/mL) was used as a positive control. Values are mean ± SD from three independent experiments. Different alphabets represent significant differences between samples (*p* < 0.05).

The mean value of neurite-bearing cells in NGF treated cells (positive control) was 22.67 ± 0.74% as shown in Fig 2. The aqueous and ethanol extracts of the *P*. *giganteus* (20 μg/mL) caused a significant (*p* < 0.05) increase in neurite-bearing cells by 4.0- and 4.7-times when compared to the control cells with medium only. *P*-coumaric, cinnamic, and oleic acids (100 μM) caused little or no neuritogenic effects on differentiating N2a cells, i.e. 8.56 ± 1.51%, 10.17 ± 1.88%, 10.06 ± 1.32%; respectively. Linoleic acid caused 20.78 ± 2.43% of N2a cells to bear neurites. Uridine (100 μM) stimulated the highest (*p* < 0.05) percentage of neurite-bearing cell (43.09 ± 4.88%), which was 1.9-times higher than that of the positive control.

Indirect measurement (chromogenic method) was performed on N2a cells treated with mushroom extracts and compounds by using the neurite outgrowth quantification assay kit (Merck Millipore). As shown in Fig 2, uridine had the highest neurite outgrowth score as indicated by the highest OD which was 0.53 ± 0.01 absorbance units (AU). This is in agreement with the counting method of neurite outgrowth, whereby the percentage of uridine-treated neurite bearing cell was also the highest, i.e. 43.09 ± 4.88%. Overall, it was concluded that the two methods can be used simultaneously, with condition that the neurite bearing score (%) must be obtained and the chromogenic method is used as a confirmatory measurement to compliment the neurite bearing score results.

### Quantification of uridine in *P*. *giganteus* extracts

**Fig 3:**
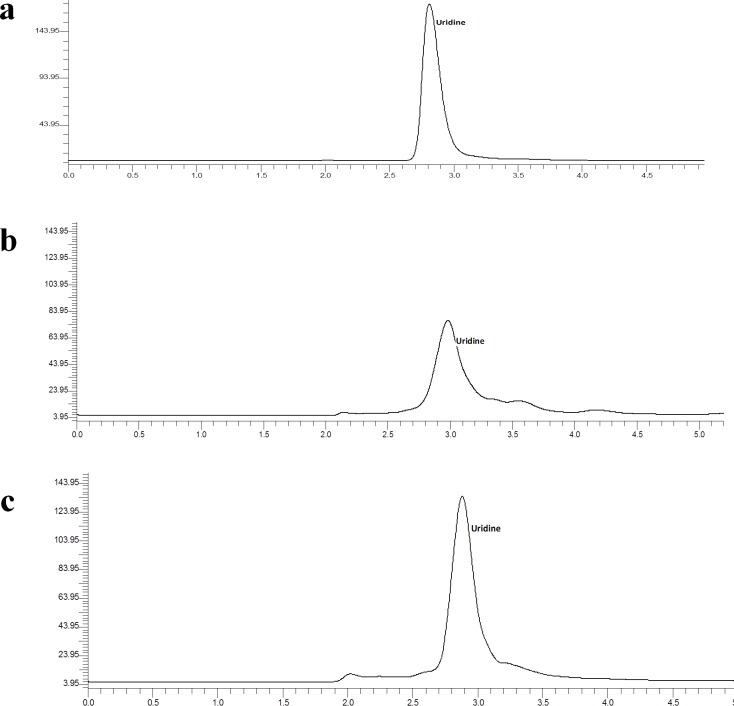
HPLC chromatogram of (a) uridine reference (Lot no.:#030M1518V), (b) ethanol extract of *P*. *giganteus*, and (c) aqueous extract of *P*. *giganteus*.

Uridine showed the highest neurite outgrowth activity; therefore the concentration of uridine was determined in the extracts of *P*. *giganteus*. Based on the calculations of the external reference uridine, aqueous extracts contained 1.80 ± 0.03% (g of compound per 100 g extract) of uridine (Table 1). On the other hand, ethanol extract yielded a lower (*p* < 0.05) amount of uridine, i.e. 1.66 ± 0.03 g/100g. The HPLC chromatograms of the mushroom extracts showed a clear peak representing uridine (Fig 3).

**Table 1:** Uridine (%, w/w) in the aqueous and ethanol extracts of *P*. *giganteus*.

Compounds	Content (%, w/w) in extract	
	Aqueous	Ethanol
Uridine	1.80 ± 0.03^a^	1.66 ± 0.03^b^

Note: (w/w) = g of compound per 100 g of mushroom extract. Different alphabets mean significant difference at *p* < 0.05.

### The effects of uridine on neurite outgrowth activity

**Fig 4:**
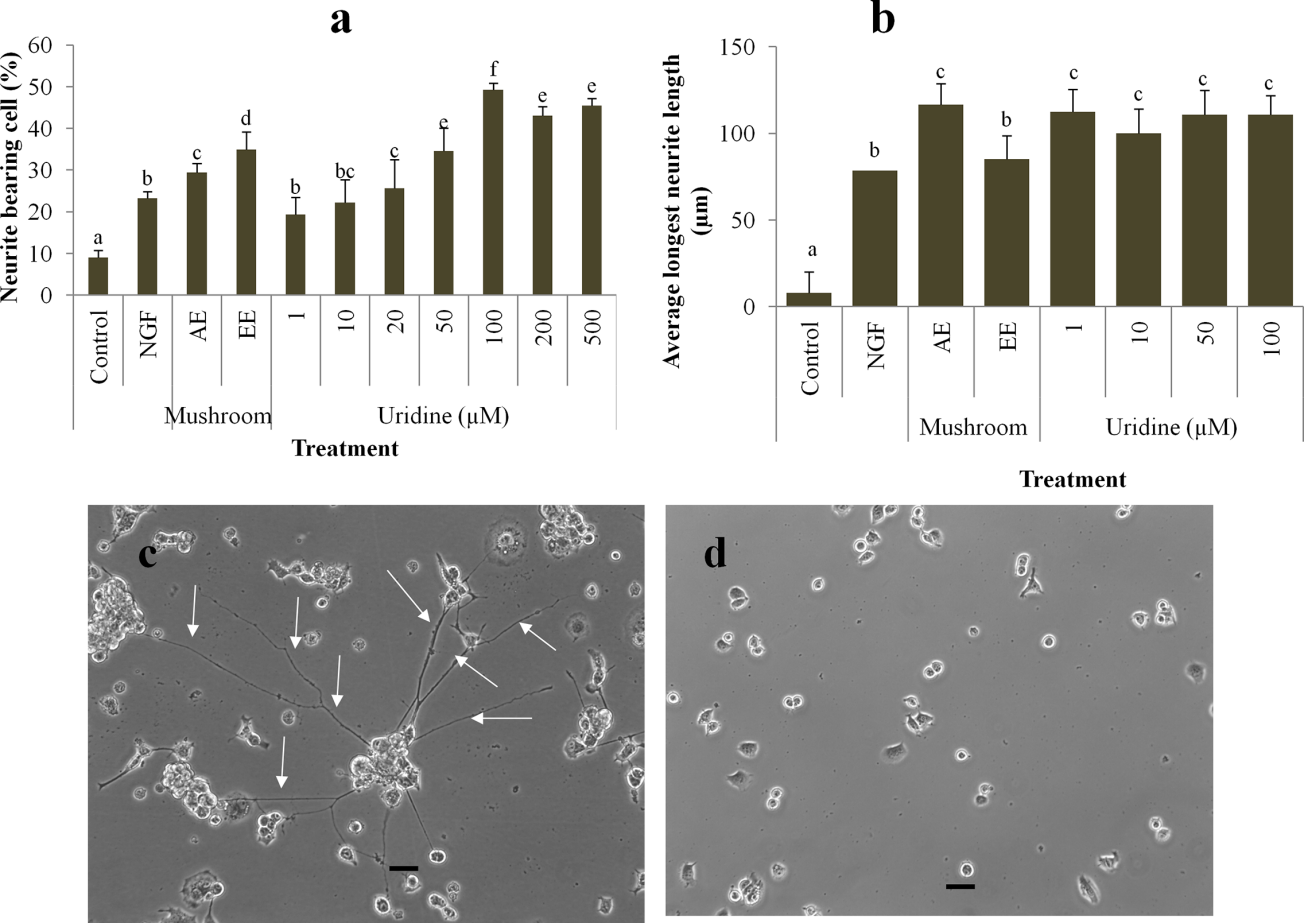
(a) The effects of different concentrations of uridine (1–500 μM) on neurite outgrowth in differentiating N2a cells. NGF (50 ng/mL) was used as positive control and medium only (vehicle) with no treatment was used as control. Aqueous and ethanol extracts (20 μg/mL) were compared to uridine. (b) The mean of the longest neurite length of N2a. Values are mean ± SD from three independent experiments. Different alphabets represent significant differences between samples (*p* < 0.01). Phase-contrast photomicrographs of (c) uridine (100μM) induced neurites as indicated by arrows and (d) negative control cells treated with only vehicle (serum-free medium). Scale bar represents 20 μm.

Taking into account that the uridine content is 1.6–1.8% (g/100 g), 20 μg/mL in ethanol and aqueous extracts would correspond to 0.32 and 0.36μg/mL of uridine; respectively. The molecular weight of uridine is 244g/mol and this would correspond to about 1.5 μM of uridine. To determine the optimum uridine concentration for neurite outgrowth activity, pure uridine at the range of 1–500 μM was tested. Treatment with uridine significantly (*p* < 0.01) increased neurite outgrowth in N2a cells in a dose dependent manner up to 100 μM (Fig 4A). One μM of uridine induced 19.4 ± 4% of neurite bearing cell which was 2 times higher than that of negative control (*p* < 0.01) and with no significant difference from the NGF control (*p* > 0.01). This means that the amount of uridine present in the mushroom extract (1.5 μM) would be sufficient to induce a significantly (*p* < 0.01) high percentage of neurite bearing cell. Uridine at 100 μM exhibited a significantly (*p* < 0.01) higher percentage in neurite-bearing cells (49.2 ± 1.6%) when compared to NGF- and mushroom extract-treated cells. Concentration of more than 100 μM did not cause additional neurite outgrowth stimulation. Therefore, the optimum uridine concentration used for the subsequent experiment was taken as 100 μM. The dose-response data was analysed to obtain the concentration of uridine that caused half-maximal neurite outgrowth stimulatory activity (EC_50_) and the value was found to be 10.5 μM.

The mean of the longest neurite length of N2a cells upon treatment with uridine was also estimated. The mean of the longest neurite length did not increase with the increasing concentrations of uridine tested (Fig 4B). The average neurite length of uridine (1 μM)-treated cells was 112.5 ± 13 μm and it was not significantly different when compared to neurite length in the 100 μM of uridine treatment group. Besides, the mean of the longest neurite length of the cells treated with *P*. *giganteus* aqueous and ethanol extracts were 116.7 ± 30 μm and 85.2 ± 13 μm; respectively. Fig 4C and 4D show the morphology of the N2a cells with neurite outgrowth after 48 h of treatment with uridine (100 μM) and vehicle (serum-free medium).

### The effects of P2Y receptor, MAPK/ERK1/2 and PI3K/Akt signaling pathway inhibitors on neurite outgrowth activity of N2a cells

**Fig 5:**
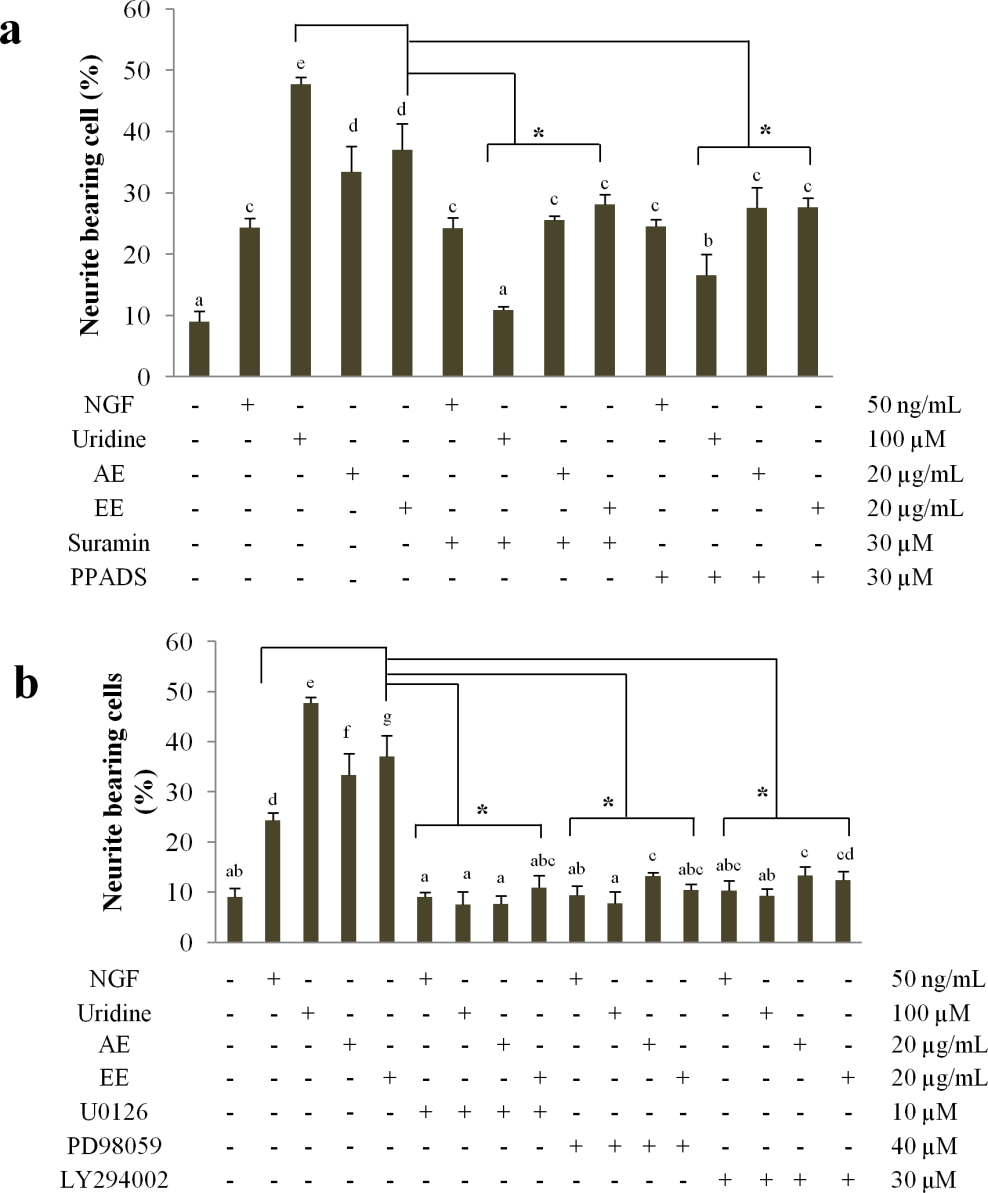
(a) The effects of two specific inhibitors of P2Y receptors (suramin and PPADS) on neurite outgrowth of differentiating N2a cells. (b) The effects of specific inhibitors of MEK/ERK and PI3K/Akt pathways (U0126 & PD98059; and LY294002, respectively) on neurite outgrowth of differentiating N2a cells. NGF (50 ng/mL) was used as positive control and medium only with no treatment was used as control. Aqueous (AE) and ethanol extracts (AE; 20 μg/mL) were tested to compare with uridine. Values are mean ± SD from three independent experiments. Different alphabets represent significant differences between samples. *p < 0.05 significantly lower relative to group without inhibitor treatment.

Specific inhibitors of key intermediates involved in neurite outgrowth signaling pathways were used to explore the mechanism of neuritogenesis in differentiating N2a cells potentiated by uridine and mushroom extracts. It was shown that neurite outgrowth induced by uridine was markedly inhibited (*p* < 0.05) by P2Y inhibitors, suramin (30 μM) and PPADS (30 μM) (Fig 5A). There is a significant decrease in the number of neuritic processes (*p* < 0.05) in the N2a cells treated with aqueous and ethanol extracts combined with either suramin or PPADS, On the contrary, both the inhibitors did not inhibit NGF-induced neurite outgrowth (Fig 5A). This could be due to a different receptor, for example the Trk family that is responsible for binding of NGF, but not P2Y receptor. MAPK/ERK1/2 inhibitors U0126 and PD98059 at the concentrations of 10 μM and 40 μM, respectively, caused inhibition effects on N2a cells. The number of differentiating N2a cells with neurite lengths double the cell diameter decreased significantly for NGF-, extracts-, and uridine-treated cells (Fig 5B). Cells pre-treated with the PI3K/Akt inhibitor, LY294002 showed no difference (*p* > 0.05) to the negative controls, with differentiated cells bearing neurites ranging only from 8.9–10.3% (Fig 5B). From these results, it is proposed that the *P*. *giganteus* extract and uridine induced neurite outgrowth on N2a cells *via* the activation of P2Y receptor. Activation of the P2Y receptor then triggered the activation of ERK1/2 and PI3K/AKt phosphorylation cascade.

### The effects of uridine on the expression of phosphorylated-(p-)ERK, p-Akt, p-MEK, p-mTOR, and p-CREB

**Fig 6:**
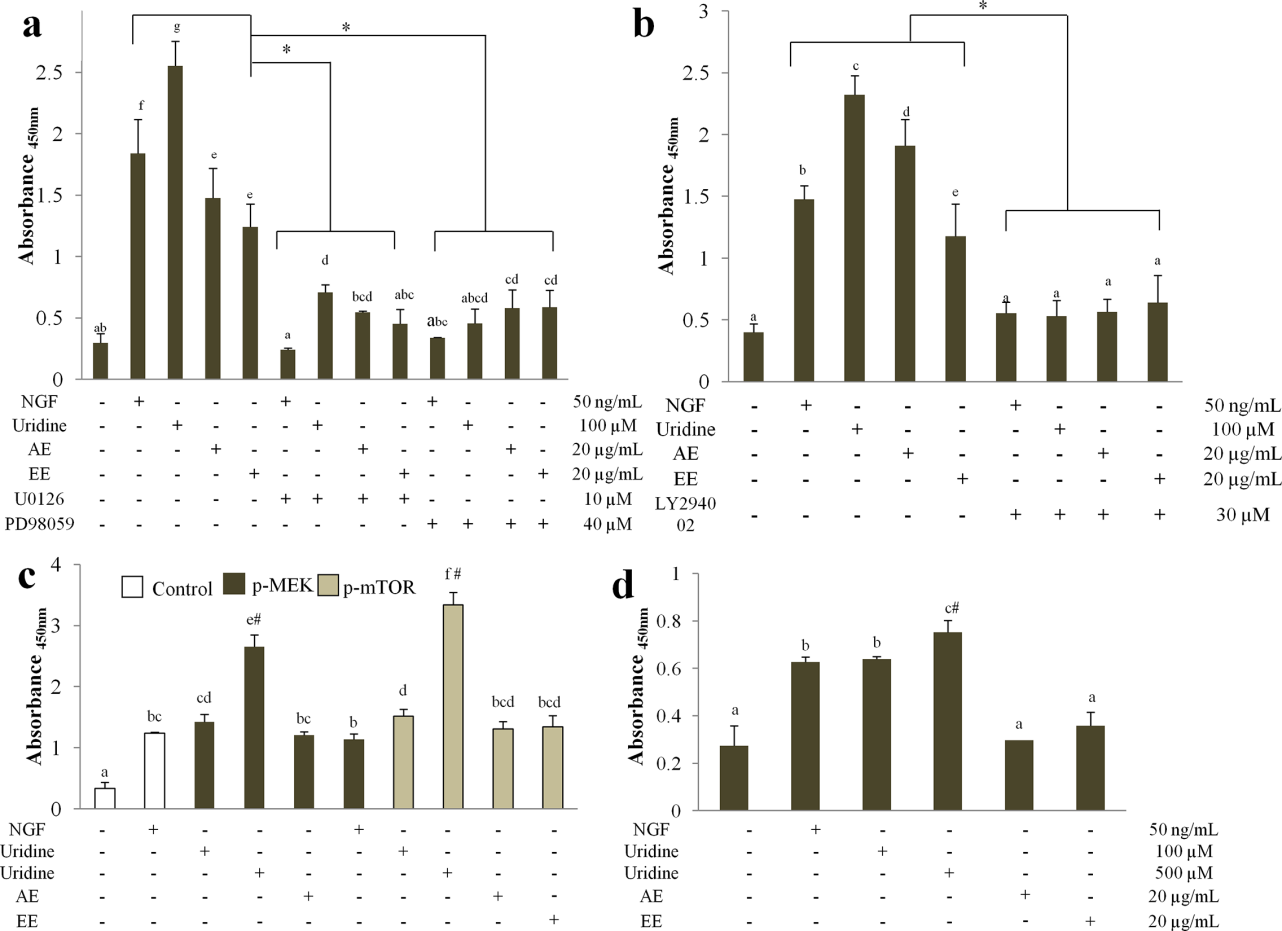
Enhancement of uridine-induced neurite outgrowth is (a) ERK-, (b) Akt-, (c) MEK- & mTOR-, and (d) CREB-dependent as evidenced by the expression of phospho-ERK1/2 (p44/p42), Akt (Thr308), MEK (Ser217/221) & mTOR (Ser2448), and CREB (Ser133) as detected using specific antibodies. U0126 and PD98059 are the inhibitors for ERK. LY294002 is the inhibitor for PI3K/Akt. Data are expressed as mean ± S.D. for n = 3. Different alphabets represent significant differences between samples (*p* < 0.05). *p < 0.05 significantly lower relative to group without inhibitor treatment. ^#^ *p* < 0.05 significantly higher relative to NGF.

Since the uridine-induced neurite outgrowth activity was found to be mediated by P2Y, MAPK/ERK1/2, and PI3K/Akt pathways, the ability of uridine to activate the specific protein kinases responsible for the pathways was tested. The phosphorylation of the downstream target proteins was measured. P-ERK activity (Fig 6A) was significantly (*p* < 0.05) enhanced by uridine when compared to the NGF-treated cells. The presence of the aqueous and ethanol extracts also triggered significantly (*p* < 0.05) higher phospho-p44/42 (Thr202/Tyr204) levels when compared to cells with medium only. Besides, uridine resulted in the highest expression of p-ERK (Fig 6A). As expected, ERK phosphorylation was significantly (*p* < 0.05) suppressed in the presence of inhibitors U0126 and PD98059. N2a cells treated with uridine also exhibited a significantly higher (*p* < 0.05) Akt (Thr308) phosphorylation when compared to the NGF control (Fig 6B). Similarly, AKT inhibitor LY294002-treated cells added with mushroom extracts, uridine, or NGF did not result in phosphorylation of Akt. Considering the above results showing that uridine significantly (*p* < 0.05) enhanced the expression of p-ERK and p-Akt, it will be crucial to evaluate whether uridine could also increase p-MEK and p-mTOR. Treatment of N2a cells with uridine caused a significant (*p* < 0.05) and sustained increase of phosphorylation of MEK and mTOR in dose dependent manner (Fig 6C). The highest phosphorylation levels of MEK and mTOR (2.65±0.2 and 3.33±0.2, respectively) were observed with 500 μM uridine. CREB is critical for activating the transcription of genes controlled by the cAMP-response element, and many of these genes may be involved in neuronal outgrowth and plasticity. Therefore, the phosphorylation of CREB was investigated. As shown in Fig 6D, treatment of N2a cells with 500 μM of uridine caused approximately 2.8 fold increase of CREB phosphorylation when compared with the vehicle group.

### The effects of uridine on the expression of GAP-43, TUBA4A, and TUBb1

**Fig 7:**
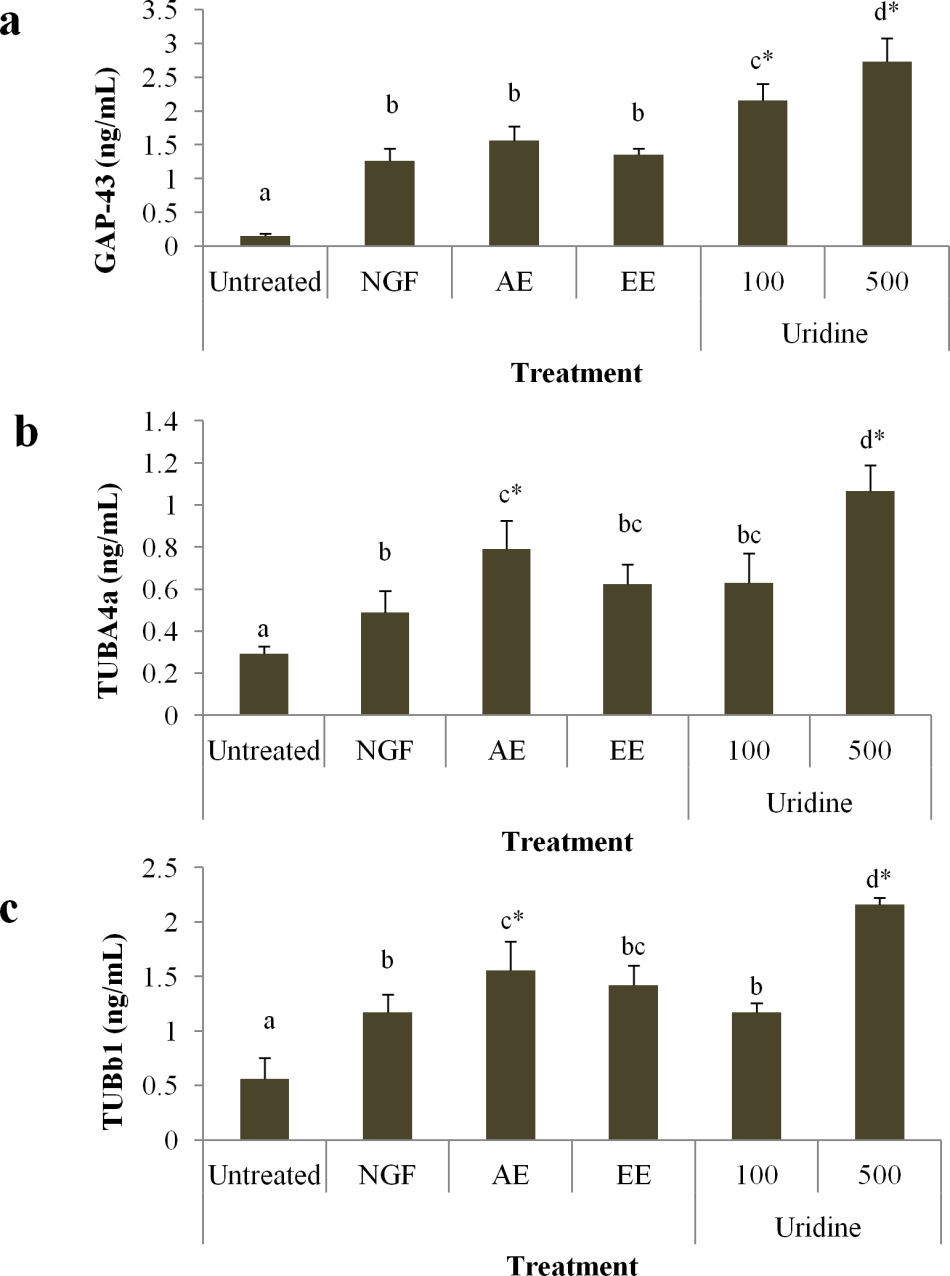
Uridine and mushroom extracts increased the activity of GAP-43 (a), Tubulin alpha 4a (b), and tubulin beta b1 (c) in N2a cells. Data are expressed as mean ± S.D. for n = 3. Different alphabets represent significant differences between samples (*p* < 0.05). *p < 0.05 significantly higher relative to NGF.

As shown in Fig 7A, uridine (100 and 500 μM) significantly (*p* < 0.05) increased total GAP-43 (0.63±0.1 and 1.07±0.1ng/mL of protein, respectively) level in a dose-dependent manner in N2a cells. The role of cytoskeleton in uridine-induced neurite outgrowth was then investigated. Tubulin a4a, the neurite outgrowth marker was evaluated in the presence of uridine. The up-regulation in tubulin a4a expression was significant (*p* < 0.05) when treated with 100 and 500 μM of uridine when compared to NGF (Fig 7B). Notably, concentration of 500μM uridine resulted in a significantly (*p* < 0.05) higher tubulin a4a expression when compared to the NGF-treated cells by 2.18-fold. Moreover, aqueous extract-treated N2a cells expressed 0.79±0.1 ng/mL of tubulin a4a, which was 1.6-fold higher than that of NGF-treated cells. Tubulin beta represents another important cytoskeleton component in neurite outgrowth. As shown in Fig 7C, the expression of tubulin beta b1 was significantly (*p* < 0.05) up-regulated by the aqueous extract of *P*. *giganteus* (1.56±0.3 ng/mL of protein). Treatment with uridine (500 μM) led to an up-regulation of tubulin beta b1 level by 1.8-fold (2.16±0.06 ng/mL of protein) when compared to NGF-treated cells.

## Discussion

Uridine has been recognised as one of the main bioactive compounds in the medicinal mushroom, *Cordyceps militaris*. The mycelial extract of *C*. *militaris* NBRC 9787 and *C*. *militaris* G81-3 were reported to contain 106.8 and 45.4 mg/kg extract of uridine [14]. More recently, a total of 0.20, 0.79, 1.50, 1.40, and 0.80 mg/g extract of uridine was detected in *Agrocybe aegerita*, *Boletus nigricans*, *Boletus fulvus*, *Tricholoma matsutake*, and *Auricularia auricular-judae*; respectively [15]. However, this study does not report any bioactivity of uridine isolated from the mushrooms. Besides, fractions F-K of the methanol extract of the “pig’s ear” mushroom, *Gomphus clavatus* demonstrated a high scavenging activity (63.8–0.1 70.3%) against 1,1-diphenyl-2-picrylhy-drazyl (DPPH) radicals. Uridine and several other compounds such as nicotinic acid and inosine were reported to be present in the fractions [16]. In this study, the dietary supplementation of *P*. *giganteus* extract would be 1.3 mg/mL by calculation.

N2a neuroblastoma cell line is derived from the mouse C1300 tumour and differentiates into a neuron-like cell that exhibits both cholinergic and adrenergic markers [17]. Further, the cells exhibit neurofilaments which belong to the class of intermediate filaments and are one of the most abundant structural proteins in axons. Our previous work has demonstrated that neurofilaments proteins was expressed in N2a cells after treatment with *P*. *giganteus* extract [9]. Therefore, we used N2a cell line as an *in vitro* model. The enhancement of neurite outgrowth and membrane synapses formed depend on the levels of three key nutrients in the brain, i.e. uridine, docosahexaenoic acid (DHA), and choline [18]. Therefore, it is thought that uridine could be beneficial to AD, a disease characterised by loss of neurite outgrowth and brain synapses [18]. Uridine is present as such in breast milk [19], but also as constituents of RNA, nucleotides (5’-UMP), and nucleotide adducts (UDP-glucose or UDP-galactose) [20]. Synthetic infant formulas are routinely fortified with uridine and uridine monophosphates (UMP). Cytidine (as cytidine triphosphate, CTP) and uridine (which is converted to UTP and then CTP) contribute to brain phosphatidylcholine and phosphatidylethanolamine synthesis *via* the Kennedy pathway [21]. In gerbils (*Meriones unguiculatus*) and humans, the primary circulating pyrimidine is uridine. Uridine readily penetrates the blood-brain barriers (BBB) and enters the brain *via* a high-affinity transporter yielding UTP which is then converted to CTP by CTP synthase. Intracellular levels of uridine triphosphate (UTP) depend on the availability of free uridine. In a study using PC12 cells, uridine significantly increased the number of neurites per cell in a dose-dependent manner after 4 days of treatment [22]. This finding was accompanied by an increase in neurite branching as well as neurofilament M and neurofilament 70. Uridine treatment also increased intracellular levels of CTP which suggests that uridine may affect neurite outgrowth by enhancing phosphatidylcholine synthesis. Since uridine enhances the production and extension of neurites, we hypothesised that increasing the availability of uridine may further promote neurite outgrowth in N2a cells.

In this study, uridine has been shown to mediate neurite outgrowth. The effect was blocked by P2Y receptor antagonists, suggesting that uridine may promote neurite outgrowth by uridine-mediated stimulation of a P2Y receptor-coupled signaling pathway. This observation is in agreement with previously reported neurotrophic effects of P2Y receptors. Exogenous uridine and its phosphorylated products, such as UMP, UDP, and UTP act as ligands for P2Y receptors which then can activate downstream protein synthesis related to neuronal differentiation. There are eight different mammalian P2Y receptor subtypes (P2Y1, 2, 4, 6, 11, 12, 13, and 14) and only P2Y2, P2Y4 and P2Y6 accept uridine nucleotides as ligands [23]. UDP and UTP have been reported previously to modulate noradrenaline release from cultured rat superior cervical ganglia [24]. In addition, the pathway involved in UTP-evoked noradrenaline release was then shown to be mediated by P2Y6 receptors *via* activation of protein kinase C [25]. Uridine has been shown to excite sensory neurons *via* P2Y2 receptors [26] and most recently, extracellular UDP-glucose has been reported to stimulate neurite outgrowth *via* the purinergic P2Y14 receptor [27].

The cAMP responsive element binding protein, CREB is a bZIP transcription factor that activates target genes through cAMP response elements. CREB is able to mediate signals from numerous physiological stimuli, resulting in regulation of a wide array of cellular responses. CREB plays a dominant regulatory role in the nervous system by promoting neuronal survival, precursor proliferation, neurite outgrowth, and neuronal differentiation in certain neuronal populations [28]. Some of the kinases involved in phosphorylating CREB at Ser133 are the MAPK and PI3K/Akt. Therefore, it is hypothesised that uridine present in the *P*. *giganteus* extracts could be metabolised in the N2a cells, and uridine along with its phosphates derivatives bind P2Y receptors and activates the MAPK and PI3K/Akt pathways, leading to the phosphorylation of the transcription factor CREB that is able to selectively activate numerous downstream genes such as the growth associated protein 43 (GAP-43) and tubulins.

There is mounting evidence supporting the fact that growing neurons express high levels of GAP-43 and that the up-regulation of GAP-43 mRNA and protein is associated with neurite outgrowth [29]. The results in this study showed that after exposure to uridine, N2a cells exhibited morphological changes and neurite formation along with up-regulation of GAP-43. This is consistent with previous findings which demonstrated that DHA significantly increased the cellular GAP-43 immunoactivity and GAP-43 content in N2a cells [30]. Dishevelled (Dvl), a cytoplasmic protein involved in the Wnt-Frizzled signaling cascade, has also been shown to interact with the cytoskeleton through modulation of GAP-43 that caused neurite outgrowth in N2a cells [31]. Claulansine F (Clau F) is a carbazole alkaloid isolated from the stem of wampee, *Clausena lansium* (Lour) Skeels [32]. Clau F was found to elevate GAP-43 expression, which in turn triggered neuritogenesis in PC12 cells. Further, 5-hydroxy-3,6,7,8,3’,4’-hexamethoxyflavone (5-OH-HxMF), which is found exclusively in the Citrus genus (particularly in the peels of sweet orange) was found to promote neurite outgrowth in PC12 cells. Accordingly, it was reported that there was a strong positive correlation of the elevated GAP-43 expression with neurite outgrowth [33]. Additionally, an increase in GAP-43 protein is associated with neuritogenesis in NGF-treated PC12 cells, as mediated by green tea polyphenols [34].

**Fig 8:**
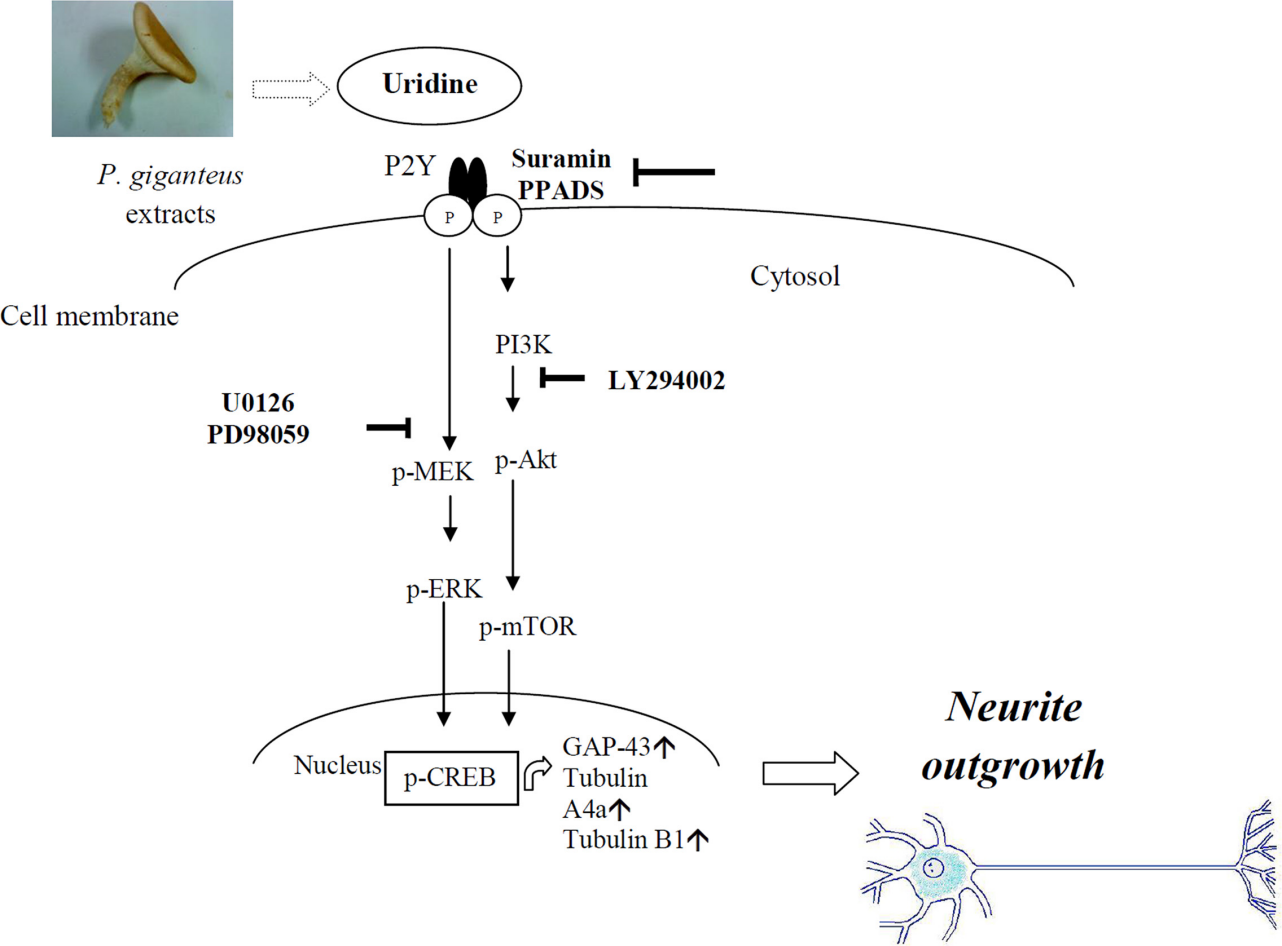
Hypothetic mechanism of uridine in promoting neurite outgrowth in N2a cells. Uridine could induce neurite outgrowth associated with expression of neuronal differentiation marker (GAP-43, tubA4a, and tubB1). Uridine stimulated CREB phosphorylation and neurite outgrowth mainly through activation of P2Y-dependent pathway. MEK/ERK1/2 and PI3K/Akt/mTOR were also partly involved in the uridine-induced neurite outgrowth. ↑ = increase.

The findings in this study clearly demonstrate that uridine promoted neurite outgrowth in differentiating N2a cells dose-dependently and the activity was regulated by the MEK/ERK and PI3K/Akt/mTOR pathways with the activation of the transcription factor CREB. The neuronal biomarkers (GAP-43, tubulin alpha 4a, and beta) in N2a cells were also significantly (*p* < 0.05) increased when treated with uridine. Taking into account that the uridine content is 1.6–1.8% (g/100g) in the basidiocarps of *P*. *giganteus*, 20 μg/mL of ethanol and aqueous extract would correspond to 0.32 and 0.36μg/mL of uridine; respectively. The molecular weight of uridine is 244g/mol and this would correspond to about 1.5 μM of uridine. In a separate experiment, the optimum pure uridine concentration on neurite outgrowth was 100 μM. Hence, by calculation the dietary supplementation of *P*. *giganteus* extract would be 1.3 mg/mL. Fig 8 shows the hypothetic mechanism of uridine in promoting neurite outgrowth in differentiating N2a cells.
